# Promoting smoking cessation in the paediatric respiratory clinic

**DOI:** 10.1007/s00431-022-04453-4

**Published:** 2022-04-12

**Authors:** Mira Osinibi, Adam Lawton, Cara Bossley, Atul Gupta

**Affiliations:** 1 King’s College Hospital, London, UK; 2 North Middlesex Hospital, London, UK; 3 Respiratory Paediatrics, Kingʼs College Hospital NHS Foundation Trust, Denmark Hill, London, UK

**Keywords:** Smoking cessation, Paediatric respiratory medicine, Carbon monoxide monitoring, Motivational interviewing

## Abstract

Exposure to tobacco smoke is harmful to children and young people (CYP). There is, to our knowledge, no published evidence quantifying the success of smoking cessation interventions targeted at both CYP and their parents or guardians in paediatric respiratory clinics. We offered 102 participants smoking cessation advice, using motivational interviewing and exhaled carbon monoxide measurements to help them quit smoking. In total, 16 of 102 participants quit smoking, with 4 lost to follow-up. A further 40 participants cut down on how much they smoked.

*Conclusion*: Formal screening questions on smoking and the provision of smoking cessation advice should form a regular part of all respiratory clinics where CYP and their parents are seen. Simple smoking cessation interventions can lead to reduced smoking in this population.

**Table:** 

**What is Known:** *• Tobacco smoking is strongly associated with significant morbidity and mortality.* *• Adolescents with chronic respiratory diseases may themselves smoke, or may have parents who do so.*	
**What is New:** *• Smoking cessation interventions are well received in paediatric respiratory clinic by patients and their families.* *• Simple smoking cessation interventions can help young people and their parents to stop smoking or cut down on smoking.*

**What is Known:**

*• Tobacco smoking is strongly associated with significant morbidity and mortality.*

*• Adolescents with chronic respiratory diseases may themselves smoke, or may have parents who do so.*

**What is New:**

*• Smoking cessation interventions are well received in paediatric respiratory clinic by patients and their families.*

*• Simple smoking cessation interventions can help young people and their parents to stop smoking or cut down on smoking.*

## Manuscript

Exposure to tobacco smoke is harmful to children and young people (CYP). In particular, it has a detrimental effect on asthma control. This is true regardless of whether the smoke is inhaled by the CYP directly or inhaled second-hand from people with whom they live. Furthermore, research has shown that not only are children of smokers more likely to smoke but that parental smoking cessation reduces the likelihood of their children smoking [1]. It is therefore worthwhile to target smoking cessation services at both our adolescent patients who smoke and their parents who accompany them to our paediatric respiratory clinics.

In 2012, the British Thoracic Society published recommendations for hospital-based smoking cessation services [2]. They gave recommendations to “seize the moment—where motivation is translated into immediate action” and reported on meta-analysis of 20 RCTs which concluded that “nursing-delivered interventions of smoking cessation significantly increase the odds of quitting”.

In recent years, the UK has cut back on funding for community smoking cessation services, and currently we are only able to refer patients who live in certain boroughs to local specialised smoking cessation clinics. We felt that our nurse-led respiratory clinics were ideally suited for smoking cessation interventions, as our team would be directly discussing the respiratory health of the CYP and negative factors that impact their health such as smokers in their immediate environment. Staff could then transition from explaining how smoking was harming CYP to measuring exhaled carbon monoxide (CO) levels (giving a rapid visual indicator of smoking-related harm) and finally offering support to stop smoking.

The King’s College Hospital Paediatric Respiratory and Cystic Fibrosis Service formally and routinely asks CYP and their caregivers about their smoking habits and offers them help to quit smoking at every clinic visit. As part of our ongoing service evaluation and improvement work, we recently analysed the impact of our smoking cessation activities within our tertiary paediatric respiratory clinics. Support with stopping smoking was offered to all adolescents and to their parents or guardians who brought them to their appointments. If they consented to participate, they received a two-part intervention consisting of motivational interviewing and exhaled carbon monoxide (CO) measurement.

Motivational interviewing is a patient-centred style of counselling that aims to “help patients explore and resolve their uncertainties about changing their behaviour” [3]. It is more effective than brief advice or usual care, and even a single short session can achieve positive results. Our motivational interviewing was delivered by paediatric respiratory clinical nurse specialists in their nurse-led outpatient clinics. Paediatric respiratory specialist nurses were trained in motivational interview techniques through a workshop run at King’s College Hospital by the Clinical Nurse Specialists in the Alcohol Misuse Team.

Exhaled carbon monoxide (CO) levels—a surrogate measure for exposure to tobacco smoke—were measured using the piCO™ Smokerlyzer® carbon monoxide breath test monitor which displays carbon monoxide levels in parts per million. If the patient had a subsequent clinic appointment within approximately 4–6 months, this measurement was repeated at the subsequent clinic appointment. The purpose of this intervention was to provide visible, quantifiable evidence that reduced exposure to tobacco smoke has a rapid and measurable effect on an individual’s health. We used pre-defined thresholds to categorise participants as non-smokers (exhaled CO of 0-6 ppm), borderline (exhaled CO of 7-9 ppm) or smokers (exhaled CO of 10 ppm or greater) [4].

In order to visually demonstrate to parents the effect of their smoking on the health of their children, we also offered CO level measurements to children 10 years and older whose parents were smokers with very high CO levels. This also allowed us to quantify a level of second-hand smoke exposure for these children.

Other substances might have been used to quantify second-hand smoke exposure—for example, urinary cotinine levels. We decided that it would be faster to collect and immediately interpret measurements of exhaled CO within the time constraints of our respiratory clinics, as well as obviating the requirement for laboratory analysis of specimens. The ability to provide adolescents and their parents with immediate point-of-care results regarding the impact of their smoking habits was also a significant advantage for us, as we could use this evidence during our motivational interviewing to encourage people to stop smoking.

In total there were 102 participants, of which 12 were adolescent patients in our respiratory clinic and 90 were the parents or guardians who brought these young people to their clinic appointment. All participants were smokers who consented to participate in motivational interview and CO measurement. More than 80% were keen or very keen to be helped with smoking cessation and expected this to be a natural part of the clinic review. Of these 102 participants, 58 were CYP or the parent of a CYP with asthma, 11 were CYP or the parent of a CYP with cystic fibrosis and 33 were CYP or the parent of a CYP with various other respiratory conditions. Initial CO measurements on declared smokers found that 57 (55.9%) had a CO above the pre-defined cut-off for smokers, and a further 29 (28.4%) had a CO that was borderline high.

Four (3.9%) of our 102 participants were lost to follow-up. Self-reported data was collected from the remaining participants, of whom 40 (39%) reported cutting down on how much they smoked and 16 (15.7%) reported that they had quit entirely.

Full data sets including smoking outcome and two CO measurements measured 4–6 months apart are available for 31 participants (Table 1). This reflects the high numbers of patients offered only one clinic appointment or offered appointments with an interval greater than 4–6 months. All CYP with available CO data who reported cutting down or quitting smoking demonstrated a fall in exhaled CO into the “non-smoker” range, suggesting a reduction in their exposure to inhaled tobacco products.

**Table 1:** Mean carbon monoxide levels before and after smoking cessation advice was given, categorised by those CYP or parents who continued, cut-down, or quit smoking

Parents (*n* = 25)			Mean carbon monoxide levels (ppm)*	
Outcome of smoking cessation intervention		Pre-counselling	Post-counselling	Change
	No change	14.4	17.1	+ 2.7
	Cut down	11.4	6.4	-5.0
	stopped	12.2	7.4	-4.8
CYP (*n* = 6)			Mean carbon monoxide levels (ppm)	
Outcome of Smoking Cessation Intervention		Pre-counselling	Post-counselling	Change
	No change	5.0	11.0	+ 6.0
	Cut down	14.0	3.0	-11.0
	Stopped	9.3	1.7	-7.7

*The pre-defined cut-off value for active smokers is a CO above 10 ppm. Pre-/post-data was available for 31/102 participants, as the majority did not require a follow-up appointment in the respiratory clinic within the audited 4–6 month period

There is, to our knowledge, no published evidence quantifying the success of smoking cessation interventions targeted at both CYP and their parents or guardians in paediatric respiratory clinics. The principal purpose of the smoking cessation promotion in our clinics is to start the journey of smoking cessation, review this repeatedly at every appointment and support smokers with the right tools and information needed to quit smoking.

Smoking cessation interventions (consisting of CO monitoring and motivational interviewing) have led to smoking reduction and smoking cessation by CYP and parents attending our tertiary paediatric respiratory clinics. The smoking cessation review that now forms part of our respiratory clinics acts to inform and advise CYP and their parents regarding smoking cessation and has helped some of our patients and their parents start their smoking cessation journeys.

Our audit of smoking cessation outcomes following routine introduction of smoking cessation activities within our paediatric respiratory clinics has demonstrated that smoking cessation can be effective in this environment and at modest financial and logistical costs to the department. Formal screening questions on smoking, and the provision of smoking cessation advice, should form a regular part of all respiratory clinics where CYP and their parents are seen. This will allow all paediatric respiratory teams to directly contribute to wider public health targets, such as the Department of Health’s efforts to drive smoking prevalence in England below 5%.

## Data Availability

Anonymised audit data available on request.

## Abbreviations

CF: Cystic fibrosis
CO: Carbon monoxide
CYP: Children and young people
